# Maternal sedentary behavior and physical activity levels in early to mid-pregnancy and obstetric outcomes: a cohort study

**DOI:** 10.1038/s41598-025-23335-x

**Published:** 2025-10-13

**Authors:** Emelie Lindberger, Fredrik Ahlsson, Henrik Johansson, Inger Sundström Poromaa, Anna-Karin Wikström

**Affiliations:** Department of Women’s and Children’s Health, Uppsala University, 751 85 Uppsala, Sweden

**Keywords:** Obstetric outcomes, Physical activity, Pregnancy, Preeclampsia, Sedentary behavior, Medical research, Epidemiology, Public health, Epidemiology

## Abstract

This Swedish cohort study aimed to evaluate if early to mid-pregnancy sedentary behavior and physical activity levels affect the risk of adverse outcomes like gestational hypertension, preeclampsia (including subtypes), gestational diabetes mellitus, labor dystocia, mode of delivery, and severe postpartum hemorrhage. Activity patterns were measured by accelerometers during seven consecutive days in 1405 women between 2016 and 2023. Logistic regression analyses adjusted for BMI, age, smoking, parity, country of birth, pre-pregnancy disease, and year of participation were performed. The accelerometer could not detect light physical activity; why only moderate and vigorous physical activity were included in the analyses. Compared with women being least sedentary, women with the highest proportion of sedentary behavior had higher adjusted odds of any preeclampsia (AOR 3.22, 95% CI 1.44–7.16), with a tendency of higher odds of severe or preterm preeclampsia (AOR 9.84, 95% CI 1.05–92.66 and AOR 5.61, 95% CI 1.04–30.23, respectively). The most physically active women had lower odds of any preeclampsia (AOR 0.22, 95% CI 0.10–0.50) than the least physically active, and the odds seemed lowest for the more severe subtypes. There were no associations with other obstetric outcomes. In conclusion, sedentary behavior and physical activity in early to mid-pregnancy might affect likelihood of preeclampsia, also after adjustment of confounders such as BMI.

**Supplementary Information:**

The online version contains supplementary material available at 10.1038/s41598-025-23335-x.

## Introduction

Sedentary behavior is defined as any waking behavior characterized by an energy expenditure ≤ 1.5 metabolic equivalents (METs), while in a sitting, reclining or lying posture^1^ and has been linked to gestational diabetes mellitus and pre- and postpartum depression^2^. The associations of sedentary behavior with other pregnancy complications, such as gestational weight gain and hypertensive disorders of pregnancy, are less clear^3^. Contrarily, physical activity during pregnancy impacts favorably on the pregnant woman and the fetus^4^. For the mother, higher level of physical activity is associated with less gestational weight gain^5^ and lower risk of gestational diabetes mellitus^6^ and preeclampsia^7,8^. It is suggested that reduced insulin resistance and improved uteroplacental blood flow might underpin these associations^9^. Furthermore, infants of physically active mothers are less likely to be born preterm^10^. Since physical activity during pregnancy has been shown to benefit most women, the American College of Obstetricians and Gynecologists (ACOG) recommends women with uncomplicated pregnancies to participate in at least 20–30 min of aerobic and strength-conditioning exercises per day^4^. Similar recommendations are also given by the World Health Organization^11^.

While there is convincing evidence of favorable effects of physical activity and reduced sedentary time on pregnancy outcomes, it is important to note that few previous studies have assessed physical activity levels, especially sedentary time, through objective methods in pregnant women^2,11–13^. The use of subjective methods, such as questionnaires or interviews might be prone to bias because levels of sedentary behavior are often underestimated and physical activity overestimated^14,15^. Hence, to confirm the findings of previous research in this field, studies assessing levels of sedentary behavior and physical activity during pregnancy through objective methods are needed.

Therefore, this study aimed to analyze the associations of objectively measured sedentary behavior and physical activity levels in early to mid-pregnancy with the following obstetric outcomes: gestational hypertension, preeclampsia (all types, severe, preterm (< 37 weeks), and term (*≥* 37 weeks) preeclampsia), gestational diabetes mellitus, labor dystocia, mode of delivery, and severe postpartum hemorrhage (PPH).

## Materials and methods

### Study design and participants

This cohort study was performed at Uppsala University hospital, Uppsala, Sweden between 2016 and 2023. The yearly birth volume of this hospital is approximately 3500–4000 deliveries. During the study period, 1713 pregnant women were recruited to the study in conjunction with either an early ultrasound scan or the second trimester ultrasound examination. The recruitment took place sequentially between May 2016 and September 2022. However, the rate of participant inclusion varied depending on the availability of recruiting research nurses and accelerometers that could be handed out. To be eligible for participation, the woman had to be 18 years or older, Swedish speaking, being pregnant in first or second trimester, having an uncomplicated pregnancy at recruitment, and having the ability to wear a gadget in the form of a wrist watch during work hours. Study participation involved carrying an accelerometer for assessment of sedentary behavior and physical activity levels for seven consecutive days. Written informed consent was obtained from all participants at inclusion. The study was approved on April 20, 2016 by the Regional Ethical Review Board in Uppsala (Dnr: 2016/142). All research was performed in accordance with relevant national and international guidelines.

### Data collection

Maternal weight was measured at the first antenatal visit and body mass index (BMI) was calculated (weight (kg)/height (m)^2^) as the weight in kilograms divided by the square of the height in meters. Information on maternal characteristics was collected by a questionnaire filled out by the woman at inclusion, and supplemented by data from the standardized antenatal and delivery medical records.

### Sedentary behavior and physical activity measurement

Sedentary behavior and physical activity levels were assessed objectively by the use of an accelerometer, either Actiwatch 2 or Actiwatch Spectrum Plus (Philips Respironics, Eindhoven, Netherlands). The Actiwatch is a monitor worn as a wristwatch that registers anteroposterior, mediolateral, and vertical movements. The Actiwatch 2 has also been validated against a waist-worn reference physical activity monitor (Actigraph GT3X) and oxygen consumption (metabolic equivalent) with adequate correlations, though the study was small and further validation was recommended^16^. The study participants were instructed to carry the accelerometer on their non-dominant wrist without interruption for seven consecutive days during all waking time, but not during engagement in water-based activities. The study participants were instructed to live their daily lives as usual. Participants with a minimum of 600 min of valid daily wear time for at least 4 days were included in the analysis. Movements were collected in 30-second epochs and reported as counts per minute (cpm)^17^ after processing by the use of Philips Actiware software (version 6.0.9). Physical activity was classified in three categories based on the following cut-off points: ≤256 cpm (sedentary), 418–720 cpm (moderate), and ≥ 721 cpm (vigorous), as per Kemp et al.^16^, and time spent in each category was calculated^17^. Due to low sensitivity and specificity of the accelerometer to characterize light physical activity (257–417 cpm),^16^ we did not include this physical activity category in our analyses. Hence, all study participants contributed with their proportion of time spent in sedentary behavior, moderate physical activity, and intense physical activity.

### Obstetric outcomes

Outcomes were gestational hypertension, preeclampsia (all types, severe, preterm, and term preeclampsia), gestational diabetes mellitus, labor dystocia, mode of delivery (spontaneous vaginal delivery, vacuum extraction, caesarean section (elective or emergency)), and severe PPH.

Gestational hypertension was identified by the International Classification of Diseases (ICD)-10 code O139 and was clinically defined as new-onset of hypertension (140/90 mmHg or above) after 20 weeks’ gestation in a previously normotensive woman.

Preeclampsia was identified by the ICD-codes O14 and O15 recorded by the obstetrician at discharge from the delivery unit. Until late 2019, preeclampsia was clinically defined as new-onset hypertension in combination with proteinuria (≥ 300 mg/24 hour, or spot urine protein/creatinine ratio ≥ 30 mg/mmol, or ≥ 1 g/L [2+] on a dipstick test) after 20 weeks’ gestation^18^. Thereafter, the definition changed to new-onset hypertension with signs and/or symptoms of end-organ dysfunction (such as injury to the kidneys (including proteinuria), liver, platelets, lungs, brain or placenta)^19^. Severe preeclampsia was identified by ICD-code O141 and clinically defined as severe hypertension (> 160/110) or severe organ dysfunction. Preterm preeclampsia was defined as preeclampsia and preterm delivery (< 37 weeks’ gestation). Term preeclampsia was defined as preeclampsia and delivery at full term (≥ 37 weeks’ gestation).

Gestational diabetes mellitus was identified by ICD-code O244 and defined as fasting plasma glucose ≥ 7.0 mmol/l or plasma glucose ≥ 9.0 mmol/l 2 h after oral intake of 75 g glucose. At Uppsala University hospital, risk factor-based screening is used. Only women identified as having an increased risk of gestational diabetes mellitus in early pregnancy (i.e. heredity for diabetes, BMI ≥ 35, or previously gestational diabetes or given birth to a large infant) or during pregnancy (e.g. random plasma glucose *≥* 8.8 mmol/L or excessive fetal growth) undergo oral glucose tolerance tests.

Labor dystocia was identified by ICD-codes O62 (no study participant had precipitate labor (O623)).

Mode of delivery was categorized into spontaneous vaginal, vacuum extraction, elective cesarean section (pre-labor), and emergency cesarean section (in labor). No woman was delivered by forceps. Data were extracted from review of medical records.

Severe PPH was defined as a bleeding ≥ 1000 ml within 24 h of delivery and was identified by review of medical records.

### Statistical methods

We used IBM SPSS Statistics version 28 to perform the statistical analyses. A nominal two-sided *p*-value < 0.05 was considered to indicate statistical significance. The proportion of time spent being sedentary and the proportion of time spent being physically active were normally distributed. The study population was divided in tertiles with respect to proportion of time spent being sedentary. The cut-off point for the most sedentary tertile was ≥ 64.5% and the cut-off point for the least sedentary tertile was ≤ 55.9%. Likewise, the cohort was divided in tertiles with respect to the proportion of time spent being physically active (sum of the proportion of time spent in moderate and vigorous physical activity). The cut-off point was ≥ 29.1% for the most physically active tertile and ≤ 21.1% for the least physically active tertile. To evaluate differences in metabolic variables between groups, we performed T-tests on continuous variables and Chi-square tests on categorical variables. Logistic regression analyses were performed to assess the association of maternal early to mid-pregnancy sedentary behavior and physical activity levels with obstetric outcomes. A directed acyclic graph (DAG)^20^ was used in the process of selecting covariates (Supplementary Fig. S1 online). Adjustments were made for first trimester BMI (kg/m^2^), age (years), smoking at first antenatal visit (yes or no), parity (nulliparous or parous), country of birth (within Europe or outside Europe), a composite of pre-pregnancy disease (yes or no), and year of study participation. The composite of pre-pregnancy diseases included inflammatory disease (for example Crohn’s disease, ulcerative colitis, and pericarditis), pre-pregnancy hypertension, hypothyroidism, diabetes mellitus (type 1 and type 2), neurological disease, chronic kidney disease, rheumatic disease, mental illness, and other severe chronic diseases. Associations are presented as odds ratios (OR) and adjusted odds ratios (AOR) with 95% confidence intervals (CI).

## Results

### Study population

Of the 1713 women recruited to this study, 308 were excluded from further analyses due to the following reasons: technical problems with analysis of physical activity data (*n* = 56), insufficient wear time (< 4 days or < 600 min of valid daily wear time) (*n* = 85), lost to follow-up (*n* = 125), multiple pregnancy (*n* = 18), abortion or miscarriage (*n* = 12), and certain data missing from pregnancy (*n* = 12). Thus, the final study population consisted of 1405 pregnant women. Out of these, 38 did not deliver their child at Uppsala University hospital why delivery data is missing. The mean maternal age was 31.5 years (standard deviation (SD) 4.4 years), and 691 (49.4%) were pregnant with their first child. The study population characteristics are presented in Table 1. The excluded cases did not differ from the final study cohort with regards to age, pre-pregnancy weight, height, parity, country of birth, and smoking status at first antenatal visit.

**Table 1:** Study population characteristics.

Variable	Whole cohort	Most sedentary tertile	Least sedentary tertile	Most physically active tertile	Least physically active tertile
n	1405	468	468	469	468
Age, years (mean ± SD)^a^	31.5 ± 4.4	31.6 ± 4.5	31.5 ± 4.2	31.4 ± 4.3	31.7 ± 4.5
Country of birth within Europe, n (%)	1269 (90.3)	411 (87.8)	435 (92.9)	435 (92.8)	415 (88.7)
Nulliparous, n (%)^b^	691 (49.4)	294 (63.1)	170 (36.5)	175 (37.5)	286 (61.4)
Smoking at first antenatal visit, n (%)^b^	111 (7.9)	32 (6.9)	43 (9.2)	40 (8.6)	31 (6.7)
Inflammatory disease, n (%)	42 (3.0)	16 (3.4)	16 (3.4)	16 (3.4)	18 (3.8)
Pre-pregnancy hypertension, n (%)	17 (1.2)	5 (1.1)	6 (1.3)	7 (1.5)	5 (1.1)
Hypothyroidism, n (%)	104 (7.4)	31 (6.6)	40 (8.5)	37 (7.9)	33 (7.1)
Diabetes mellitus type 1 and type 2, n (%)	15 (1.1)	7 (1.5)	2 (0.4)	3 (0.6)	7 (1.5)
Neurological disease, n (%)	10 (0.7)	1 (0.2)	6 (1.3)	7 (1.5)	1 (0.2)
Chronic kidney disease, n (%)	1 (0.1)	0 (0.0)	0 (0.0)	0 (0.0)	0 (0.0)
PCOS, n (%)	26 (1.9)	10 (2.1)	10 (2.1)	10 (2.1)	9 (1.9)
Rheumatic disease, n (%)	20 (1.4)	6 (1.3)	7 (1.5)	7 (1.5)	6 (1.3)
Previous depression or anxiety, n (%)	203 (14.4)	76 (16.2)	64 (13.7)	63 (13.4)	73 (15.6)
Ongoing depression, n (%)	117 (8.3)	40 (8.5)	41 (8.8)	46 (9.8)	36 (7.7)
Ongoing anxiety, n (%)	104 (7.4)	37 (7.9)	36 (7.7)	37 (7.9)	35 (7.5)
Autism, n (%)	5 (0.4)	2 (0.4)	0 (0.0)	0 (0.0)	2 (0.4)
ADHD, n (%)	24 (1.7)	8 (1.7)	7 (1.5)	6 (1.3)	7 (1.5)
Bipolar disorder, n (%)	16 (1.1)	4 (0.9)	4 (0.9)	6 (1.3)	5 (1.1)
Eating disorder, n (%)	18 (1.3)	5 (1.1)	4 (0.9)	5 (1.1)	4 (0.9)
Other severe chronic disease, n (%)	53 (3.8)	6 (1.3)	23 (4.9)	27 (5.8)	10 (2.1)

*ADHD* attention deficit hyperactivity disorder, *PCOS* polycystic ovary syndrome, *SD* standard deviation. ^a^Data missing in 1 individual. ^b^Data missing in 5 individuals.

### Daily sedentary behavior and physical activity patterns

Sedentary behavior and physical activity levels were measured between gestational weeks 8‒23. Approximately two thirds of the study population (*n* = 966) were recruited in conjunction with an early ultrasound scan (median gestational length at start of measuring period 90 days, interquartile range (IQR) 5 days). The rest (*n* = 428) was recruited at the second trimester ultrasound examination (median gestational length at start of measuring period 133 days, IQR 7 days). The measuring period ranged 4‒7 days (mean 6.7 days) and the mean daily awake wear time was 15.7 h (SD 1.1 h).

### Maternal metabolic variables in relation to sedentary behavior and physical activity levels

Metabolic variables in relation to sedentary behavior and physical activity levels are presented in Table 2. The number of cases with missing data is presented in Supplementary Table S1 online. Women in the most sedentary tertile had higher first trimester body weight, higher first trimester BMI, and were more commonly obese compared with women in the least sedentary tertile. Higher diastolic blood pressure during first trimester was seen among the most sedentary women compared with the least sedentary, although the difference was very small (about 1 mmHg). When comparing the most physically active tertile with the least physically active tertile, the same variables differed between the groups; the most physically active women had lower first trimester body weight, lower first trimester BMI, were less frequently obese, and had a slightly lower diastolic blood pressure during first trimester compared with the least physically active women.

**Table 2:** Metabolic variables in relation to sedentary behavior and physical activity levels.

Variable	Most sedentary tertile (*n* = 468)	Least sedentary tertile (*n* = 468)	*p*	Most physically active tertile (*n* = 469)	Least physically active tertile (*n* = 468)	*p*
Weight at first antenatal visit, kg (mean ± SD)	**71.1 ± 15.5**	**68.8 ± 13.1**	**0.015**	**68.5 ± 12.5**	**71.8 ± 16.0**	**< 0.001**
BMI at first antenatal visit, kg/m^2^ (mean ± SD)	**25.4 ± 5.1**	**24.6 ± 4.4**	**0.006**	**24.5 ± 4.2**	**25.7 ± 5.3**	**< 0.001**
Overweight (BMI 25.0–29.9 kg/m^2^) at first antenatal visit, n (%)	133 (28.6)	118 (25.4)	0.276	124 (26.7)	140 (30.1)	0.253
Obesity (BMI ≥ 30.0 kg/m^2^) at first antenatal visit, n (%)	**76 (16.3)**	**52 (11.2)**	**0.023**	**51 (11.0)**	**77 (16.6)**	**0.014**
Gestational weight gain, kg (mean ± SD)	13.3 ± 5.1	13.2 ± 4.8	0.625	13.2 ± 4.8	13.2 ± 5.1	0.995
Systolic blood pressure first trimester, mmHg (mean ± SD)	113 ± 10	113 ± 10	0.622	113 ± 10	113 ± 10	0.803
Diastolic blood pressure first trimester, mmHg (mean ± SD)	**69 ± 9**	**68 ± 8**	**0.003**	**68 ± 8**	**69 ± 9**	**0.003**
Systolic blood pressure at 36 weeks’ gestation, mmHg (mean ± SD)	120 ± 13	120 ± 12	0.653	120 ± 12	120 ± 13	0.471
Diastolic blood pressure at 36 weeks’ gestation, mmHg (mean ± SD)	76 ± 11	75 ± 10	0.340	75 ± 10	76 ± 11	0.239

Bold text indicates significant results. Data were analyzed using t tests (continuous variables) and Chi-square tests (categorical variables). *BMI* body mass index,* SD* standard deviation.

### Frequencies of obstetric outcomes in relation to sedentary behavior and physical activity levels

The frequencies of adverse obstetric outcomes across groups defined by sedentary behavior and physical activity levels are presented in Tables 3 and 4, respectively. The number of cases with missing data is presented in Supplementary Table S1 online.

**Table 3:** Associations of maternal early to mid-pregnancy sedentary behavior with adverse obstetric outcomes.

Adverse outcome	Most sedentary tertile (*n* = 453)	Least sedentary tertile (*n* = 456)	Most sedentary tertile versus least sedentary tertile (reference)					
			Unadjusted model			Adjusted model^a^		
	*n* (%)	*n* (%)	OR	CI	*p*	OR	CI	*p*
Gestational hypertension	18 (4.0)	17 (3.7)	1.07	0.54–2.10	0.848	0.87	0.39–1.94	0.731
Preeclampsia (all)	29 (6.4)	11 (2.4)	**2.77**	**1.37–5.61**	**0.005**	**3.22**	**1.44–7.16**	**0.004**
Severe preeclampsia	6 (1.3)	1 (0.2)	6.11	0.73–50.93	0.095	**9.84**	**1.05–92.66**	**0.046**
Preterm preeclampsia	9 (2.0)	2 (0.4)	4.60	0.99–21.42	0.052	**5.61**	**1.04–30.23**	**0.045**
Term preeclampsia	20 (4.4)	9 (2.0)	**2.29**	**1.03–5.09**	**0.041**	2.40	0.97–5.98	0.059
Gestational diabetes mellitus	19 (4.2)	7 (1.5)	**2.81**	**1.17–6.75**	**0.021**	2.50	0.90–6.95	0.078
Elective caesarean section	26 (5.7)	30 (6.6)	0.87	0.50–1.49	0.599	0.88	0.46–1.67	0.687
Severe PPH	46 (10.4)	34 (7.7)	1.40	0.88–2.22	0.158	1.05	0.60–1.81	0.875
Labor dystocia^b^	98 (23.1)	67 (15.7)	**1.58**	**1.12–2.23**	**0.009**	0.89	0.58–1.36	0.595
Spontaneous vaginal delivery^b^	346 (81.0)	365 (85.7)	0.71	0.50–1.03	0.069	1.17	0.73–1.81	0.485
Vacuum extraction^b^	34 (9.0)	26 (6.1)	1.37	0.80–2.33	0.247	0.78	0.41–1.47	0.440
Emergency caesarean section^b^	47 (11.0)	35 (8.2)	1.38	0.87–2.19	0.168	0.90	0.52–1.57	0.719

Data are odds ratios (OR) with 95% confidence interval (CI). Bold text indicates significant results. Data were analyzed using logistic regression models. *BMI* body mass index, *PPH* postpartum hemorrhage. ^a^Adjustments in the model: first trimester BMI, age, smoking at first antenatal visit, parity, maternal country of birth, pre-pregnancy disease, and year of study participation. ^b^Only including women not undergoing elective caesarean section (*n* = 1278).

**Table 4:** Associations of maternal early to mid-pregnancy physical activity levels with adverse obstetric outcomes.

Adverse outcome	Most physically active tertile (*n* = 456)	Least physically active tertile (*n* = 452)	Most physically active tertile versus least physically active tertile (reference)					
			Unadjusted model			Adjusted model^a^		
	*n* (%)	*n* (%)	OR	CI	*p*	OR	CI	*p*
Gestational hypertension	17 (3.7)	15 (3.3)	1.13	0.56–2.29	0.738	1.72	0.72–4.10	0.223
Preeclampsia (all)	10 (2.2)	32 (7.1)	**0.29**	**0.14–0.61**	**< 0.001**	**0.22**	**0.10–0.50**	**< 0.001**
Severe preeclampsia	1 (0.2)	9 (2.0)	**0.11**	**0.01–0.86**	**0.035**	**0.08**	**0.01–0.66**	**0.019**
Preterm preeclampsia	1 (0.2)	9 (2.0)	**0.11**	**0.01–0.86**	**0.035**	**0.08**	**0.01–0.71**	**0.023**
Term preeclampsia	9 (2.0)	23 (5.1)	**0.38**	**0.17–0.82**	**0.014**	**0.31**	**0.13–0.76**	**0.010**
Gestational diabetes mellitus	8 (1.8)	18 (4.0)	0.43	0.19–1.00	0.050	0.48	0.18–1.33	0.159
Elective caesarean section	30 (6.6)	25 (5.5)	1.20	0.70–2.08	0.509	1.68	0.86–3.28	0.126
Severe PPH	37 (8.4)	48 (10.9)	0.75	0.48–1.17	0.207	1.08	0.62–1.86	0.792
Labor dystocia^b^	71 (16.7)	97 (22.9)	**0.69**	**0.49–0.97**	**0.033**	1.12	0.73–1.72	0.596
Spontaneous vaginal delivery^b^	366 (85.9)	345 (80.8)	**1.45**	**1.00–2.09**	**0.045**	0.89	0.57–1.39	0.603
Vacuum extraction^b^	22 (5.8)	33 (8.8)	0.64	0.36–1.11	0.112	1.21	0.61–2.40	0.586
Emergency caesarean section^b^	38 (8.9)	49 (11.5)	0.76	0.48–1.18	0.219	1.11	0.65–1.92	0.696

Data are odds ratios (OR) with 95% confidence interval (CI). Bold text indicates significant results. Data were analyzed using logistic regression models. *BMI* body mass index, *PPH* postpartum hemorrhage. ^a^Adjustments in the model: first trimester BMI, age, smoking at first antenatal visit, parity, maternal country of birth, pre-pregnancy disease, and year of study participation. ^b^Only including women not undergoing elective caesarean section (*n* = 1278).

### Sedentary behavior and likelihood of obstetric outcomes

Women in the most sedentary tertile had higher adjusted odds of preeclampsia (all types) compared with the least sedentary (AOR 3.22, CI 1.44–7.16, *p* = 0.004) (Table 3). The adjusted odds were also increased for severe preeclampsia (AOR 9.84, CI 1.05–92.66, *p* = 0.046), and preterm preeclampsia (AOR 5.61, CI 1.04–30.23, *p* = 0.045) among the most sedentary women (Table 3). The adjusted odds of gestational hypertension, term preeclampsia, gestational diabetes mellitus, elective caesarean section, labor dystocia, spontaneous vaginal delivery, vacuum extraction, emergency caesarean section, and severe PPH did not differ between the most and least sedentary tertiles (Table 3).

### Physical activity levels and likelihood of obstetric outcomes

There was a 78% decrease in the adjusted odds of preeclampsia (all types) in the most compared with the least physically active tertile (AOR 0.22, CI 0.10–0.50, *p* < 0.001) (Table 4). Additionally, the most physically active women had lower adjusted odds of severe preeclampsia (AOR 0.08, CI 0.01–0.66, *p* = 0.019), preterm preeclampsia (AOR 0.08, CI 0.01–0.71, *p* = 0.023), and term preeclampsia (AOR 0.31, CI 0.13–0.76, *p* = 0.010) (Table 4). We found no difference in the adjusted odds of gestational hypertension, gestational diabetes mellitus, elective caesarean section, labor dystocia, spontaneous vaginal delivery, vacuum extraction, emergency caesarean section, and severe PPH between the most and least physically active tertiles (Table 4).

## Discussion

In the present study an increased likelihood of preeclampsia, particularly the severe subtypes severe preeclampsia and preterm preeclampsia, was found among women who spent a high proportion of time in sedentary behavior in early to mid-pregnancy. In contrast, women who spent a high proportion of time in physical activity had a lower likelihood of preeclampsia, including all subtypes.

We found that a high level of sedentary behavior was associated with preeclampsia. According to a recent systematic review, few previous studies have investigated the relation between sedentary behavior and hypertensive disorders of pregnancy including preeclampsia, and the results of the previous studies are divergent^2^. The findings from a large US based study including 1240 Hispanic women are not consistent with ours; the study reports no association of early pregnancy sedentary behavior with preeclampsia^21^. However, the study assessed sedentary behavior by the use of a questionnaire, which decreases the reliability of the results^22^.

Although our results showed an association between sedentary behavior and preeclampsia, there was no association between sedentary behavior and gestational hypertension. This is in line with a previous study reporting no association between accelerometer measured sedentary time and diastolic blood pressure in 206 pregnant US women^23^. On the contrary, a Chinese study reports that pregnant women with a persistent sedentary occupation not allowing them to move around (such as sewing operators) have an increased risk of gestational hypertension compared with women having a sedentary work but with the opportunity to move (such as secretaries)^24^. Hence, the literature on the relation between maternal sedentary behavior and hypertensive disorders of pregnancy is inconsistent, and the associations with gestational hypertension and preeclampsia are not coherent.

This study demonstrated a decreased likelihood of preeclampsia among the most physically active women. This is consistent with previous research showing an inverse association of physical activity in early pregnancy with preeclampsia^7,8^. The mechanisms by which physical activity might influence the risk of preeclampsia are unclear. The pathophysiology of preeclampsia is largely unknown, but the condition is characterized by placental abnormalities, endothelial dysfunction, oxidative stress, and inflammation^25^. Physical activity has been proposed to enhance growth and vascularization of the placenta, improve endothelial function, reduce oxidative stress, decrease arterial stiffness, and improve immune and inflammatory responses^26^. Thus, physical activity seems to have an impact on several of the pathophysiological mechanisms that have been linked to preeclampsia. Our finding, that sedentary behavior and physical activity are related to an obstetric outcome involving dysfunction of the cardiovascular system is reasonable. Reduced sedentary time and increased physical activity are important non-pharmacological interventions to prevent hypertension and cardiovascular disease in non-pregnant individuals^27^.

Our results demonstrated no associations of sedentary behavior and physical activity levels with mode of delivery in the adjusted regression analyses. These results are in line with those of a Spanish study evaluating accelerometer assessed early second trimester sedentary time and physical activity levels with birth outcomes^28^. The study included 94 pregnant women with a mean age of 32.9 years. Sedentary time and physical activity recordings were performed during seven consecutive days. The study demonstrates associations of sedentary time with umbilical blood gas parameters (higher partial pressure carbon dioxide and acidic pH), and of physical activity levels with arterial cord blood oxygen saturation and Apgar score. However, no associations are reported of sedentary behavior and physical activity levels with mode of delivery. Of note is that the Spanish cohort had a higher frequency of caesarean section (24.0%) than our study cohort (16.2%).

This study had limitations and strengths. A limitation was the observational study design, which only enabled assessment of associations and not causality. It is possible that unmeasured confounders were present that could explain high levels of sedentary behavior, low levels of physical activity, and preeclampsia. For example, obstetric or medical conditions (other than those we controlled for) leading to increased sedentary behavior and decreased physical activity might also lead to increased risk of preeclampsia development. Besides underlying medical or developing obstetric conditions, socioeconomic factors such as education and income could possibly influence the association. Furthermore, a large proportion of participants were not included in the analyses due to non-compliance, difficulties with the device output or loss to follow up. This study might not have had enough power to detect small differences, as may be the case for the absent relationship between sedentary behavior and gestational diabetes in our study population. Power is indeed relevant as many obstetric outcomes are rare; in fact, some of the rarer outcomes were not even addressed in this study. Limitations also included that the activity pattern was measured at one occasion only and that the measurement period was short in relation to the length of the pregnancy. A longer measurement period would have increased the reliability of the measures, but might instead have decreased the feasibility of the study. Since the accelerometer was not worn during water-based activities, it is possible that some participants were more physically active than recorded. However, this limitation is unlikely to have had a substantial impact on the overall results, as it is reasonable to assume that only a small proportion of participants engaged in such activities to a meaningful extent. Moreover, light activity was not included in the analyses due to low sensitivity and specificity of the Actiwatch to characterize it.^16^ The exact levels of moderate and vigorous activity should also be interpreted with some caution, since the cut-off points suggested by Kemp et al. were determined in a lab setting^16^. Further research is needed to validate the ability of the Actiwatch to measure waking movement behavior in free-living settings. A major strength was the use of accelerometers that measured sedentary behavior and physical activity levels objectively^22^. Lastly, the length of the measuring period (on average 6.7 days) enabled assessment of both weekday and weekend activity patterns.

## Conclusion

We suggest that the clinical implication of this study’s results is that increased sedentary behavior in early to mid-pregnancy might indicate an increased likelihood of development of preeclampsia, and that higher physical activity levels might indicate a decreased likelihood of this complication. The possible predictive value of sedentary behavior and physical activity measurement in early to mid-pregnancy as a tool for risk assessment of preeclampsia needs to be explored by future studies.

## Supplementary Information

Below is the link to the electronic supplementary material.

Supplementary Material 1

## Data Availability

The datasets generated during and/or analyzed during the current study are available from the corresponding author on reasonable request.
