# Supplementary material for: Maternal sedentary behavior and physical activity levels in early to mid-pregnancy and obstetric outcomes: a cohort study
Source: Sci Rep. 2025 Oct 13;15:35575. doi: 10.1038/s41598-025-23335-x (PMC12518509; doi:10.1038/s41598-025-23335-x)
Supplement: Supplementary file 1 — Supplementary Material 1 [file 41598_2025_23335_MOESM1_ESM.pdf]

Supplementary information file

Maternal sedentary behavior and physical activity levels in early to mid-pregnancy and obstetric outcomes: A cohort study

Emelie Lindberger MD PhD[1], Fredrik Ahlsson MD professor[1], Henrik Johansson PhD[1], Inger Sundström Poromaa MD professor[1], Anna-Karin Wikström MD professor[1]

[1] Department of Women’s and Children’s Health, Uppsala University, 751 85 Uppsala, Sweden

Supplementary Figure S1. Directed acyclic graph (DAG) used in the process of selecting covariates.

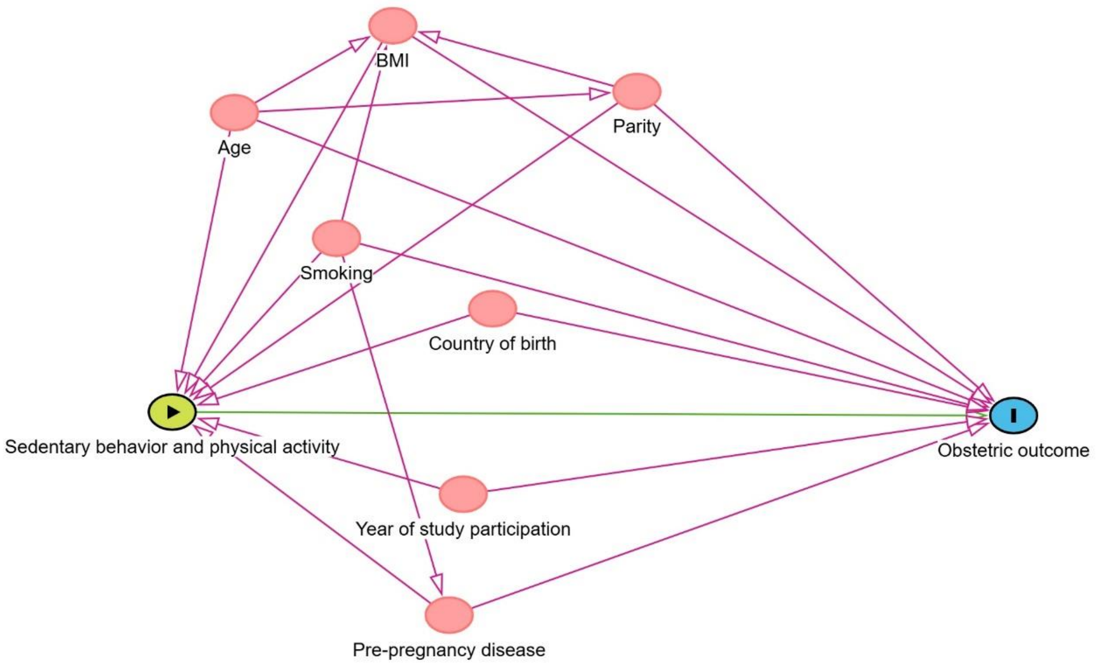

Supplementary Table S1. Overview of missing data across subgroups.

**Subgroup**	**Most sedentary tertile (n =468)**	**Least sedentary tertile (n =468)**	**Most physically active tertile (n =469)**	**Least physically active tertile (n =468)**
Cases with missing data, n (%)				
Weight at first antenatal visit	3 (0.6)	3 (0.6)	4 (0.9)	3 (0.6)
BMI at first antenatal visit	3 (0.6)	4 (0.9)	5 (1.1)	3 (0.6)
Gestational weight gain	195 (41.7)	116 (24.8)	119 (25.4)	202 (43.2)
Systolic blood pressure first trimester	15 (3.2)	10 (2.1)	10 (2.1)	17 (3.6)
Diastolic blood pressure first trimester	17 (3.6)	9 (1.9)	9 (1.9)	18 (3.8)
Systolic blood pressure at 36 weeks' gestation	143 (30.6)	57 (12.2)	57 (12.2)	150 (32.1)
Diastolic blood pressure at 36 weeks' gestation	142 (30.3)	56 (12.0)	56 (11.9)	149 (31.8)
Gestational hypertension [a]	0 (0.0)	0 (0.0)	0 (0.0)	0 (0.0)
Preeclampsia (all) [a]	0 (0.0)	0 (0.0)	0 (0.0)	0 (0.0)
Severe preeclampsia [a]	0 (0.0)	0 (0.0)	0 (0.0)	0 (0.0)
Preterm preeclampsia [a]	0 (0.0)	0 (0.0)	0 (0.0)	0 (0.0)
Term preeclampsia [a]	0 (0.0)	0 (0.0)	0 (0.0)	0 (0.0)
Gestational diabetes mellitus [a]	0 (0.0)	0 (0.0)	0 (0.0)	0 (0.0)
Elective caesarean section [a]	0 (0.0)	0 (0.0)	0 (0.0)	0 (0.0)
Severe PPH [a]	12 (2.6)	14 (3.1)	14 (3.1)	11 (2.4)
Labor dystocia [b]	3 (0.7)	7 (1.6)	8 (1.9)	3 (0.7)
Spontaneous vaginal delivery [b]	0 (0.0)	0 (0.0)	0 (0.0)	0 (0.0)
Vacuum extraction [b]	51 (11.9)	42 (9.9)	46 (10.8)	53 (12.4)
Emergency caesarean section [b]	0 (0.0)	0 (0.0)	0 (0.0)	0 (0.0)

[a] Only including women delivering at Uppsala University hospital (n =1367)

[b] Only including women not undergoing elective caesarean section (n =1278)

BMI, body mass index; PPH, postpartum hemorrhage
